# The importance of parental beliefs and support for pedometer-measured physical activity on school days and weekend days among Canadian children

**DOI:** 10.1186/1471-2458-13-1132

**Published:** 2013-12-05

**Authors:** Kerry A Vander Ploeg, Stefan Kuhle, Katerina Maximova, Jonathan McGavock, Biao Wu, Paul J Veugelers

**Affiliations:** 1 School of Public Health, University of Alberta, Edmonton Alberta, Canada; 2 Departments of Pediatrics and Obstetrics & Gynecology, Dalhousie University, Halifax, Nova Scotia, Canada; 3 Department of Pediatrics and Child Health, University of Manitoba, Winnipeg, Manitoba, Canada

**Keywords:** Physical activity, Pedometer, Childhood obesity, Public health, Health promotion

## Abstract

**Background:**

Parental influences are essential to the behaviours and physical activity of their children. Our study aimed to determine if parental beliefs and support are associated with children’s pedometer measured physical activity levels on school days and weekend days.

**Methods:**

In the spring of 2009 and 2011, we analyzed cross-sectional data from 1,355 grade five students and parents in 30 schools in Alberta, Canada. Parents reported how much they care about exercising, how much they encourage their child to be physically active, and how frequently they engage in physical activities with their child. Physical activity was assessed from step counts obtained from time-stamped pedometers collected over nine consecutive days.

**Results:**

Increased parental encouragement was positively associated with boys’ and girls’ physical activity on school days (Boys: beta = 1373, 95% CI: 606, 2139; Girls: beta = 632, 95% CI: 108, 1155) and girls’ physical activity on weekend days (beta = 997, 95% CI: 130, 1864). Increased parental care was positively associated with boys’ physical activity on weekend days (beta = 1381, 95% CI: 85, 2676). Increased parental support and engagement was associated with an additional 632–1381 steps/day for children in this study.

**Conclusions:**

Parental care, encouragement and engagement are associated with physical activity levels of children 10–11 years of age. Policy makers and researchers should consider the importance of targeting parents when designing strategies to promote physical activity in children. This is particularly relevant to weekends and holidays when children’s activity levels are low.

## Background

Regular physical activity is associated with an array of health benefits for children [1]. Unfortunately, most children in Canada are not sufficiently active. Currently, only 7% of boys and 3% of girls accumulate the recommended 13,500 steps per day at least 6 days a week [2]. We have previously demonstrated that these trends are significantly worse on weekend days, relative to weekdays [3]. These trends are concerning as low levels of physical activity during childhood contribute to obesity and comorbid conditions in adulthood [4]. As such, increasing children’s physical activity levels, particularly on weekend days, continues to be a priority for improving child health outcomes.

It is well established that parental influences are essential to the behaviours and physical activity of their children [5-7]. Children’s physical activity levels have been shown to be greater when parents are active, encourage them to be active, and engage in activities with them [6,8-11]. Reviews demonstrate that childhood is an important time to establish healthy behaviours given that behaviours established and practiced in childhood track into adulthood [12,13]. However, as children spend substantial time at school, parents may have more opportunities to influence children’s physical activity on weekend days than on school days. To our knowledge, the importance of parental beliefs and support as a correlate of children’s weekend day physical activity has never been studied.

To address this knowledge gap, we relied on recently collected cross-sectional data to test the hypothesis that parental beliefs and support for physical activity are positively associated with physical activity levels in grade five students on school and weekend days. We also hypothesized that this association is distinct for school days and weekend days.

## Methods

### Study design/setting

Raising healthy Eating and Active Living Kids in Alberta (REAL Kids Alberta) is a population-based study of grade five children and their parents in the Canadian province of Alberta. In 2008, the REAL Kids Alberta survey included 148 randomly selected schools from across Alberta as described elsewhere [14]. In 2009, we selected and invited a convenient sample of 20 of the 148 schools, located in the city of Edmonton and surrounding areas, to participate in an additional survey that included objective measures of physical activity. We also invited grade five students from 10 schools participating in the Alberta Program Promoting active Living and healthy Eating in Schools (APPLE Schools) [15]. We repeated data collection among grade five students and parents in 2011 from the same schools that participated in 2009. In 2009 and 2011 combined, we sent 2,502 parent consent forms and surveys home with students for their parents to complete and return to school; 2,045 (81.7%) home surveys were returned, 2,028 (99.2%) students received parental consent to participate. Student assent to participate was obtained from 1,991 (98.2%) children, and 1,977 (99.3%) children completed student surveys, resulting in an overall participation rate of 79.0%. Pedometers were distributed to the 1,991 students with parent consent and student assent, 1,783 pedometers were returned, providing crude hourly step counts; 210 (10.5%) pedometers were lost or malfunctioned. The Health Research Ethics Board of the University of Alberta approved this study, including data collection and parental informed consent forms.

### Assessment of parental beliefs and support

The parent survey included three validated questions related to beliefs and support that were adapted from the activity-related parenting practices scale by Davison et al. [16]. These included: 1) how much do you personally care about staying fit and exercising (a little bit; quite a lot; very much); 2) to what extent do you encourage your grade five child to be physically active (a little bit; quite a lot; very much); and 3) how often do you or another parent/guardian usually engage in physical activity together with your child (less than 1 time/week; 1–3 times/week; 4 or more times/week). The questionnaires we used are available on the project’s website: http://www.REALKidsAlberta.ca.

### Assessment of physical activity

We used Omron HJ-720ITC (Ontario, Canada) pedometers to measure physical activity objectively. This pedometer records steps hourly, automatically resets at midnight, and can store data for 42 days. Further rationale for selecting this pedometer are described in detail elsewhere [3]. The accuracy and validity of the Omron pedometer has been demonstrated under various conditions [17-19]. Students wore pedometers for nine consecutive days on their right hip directly in line with their right knee during all waking hours unless showering, swimming, or taking part in activities in which an adult deemed it unsafe to wear. Pedometer recordings from the first and ninth days were not considered as, on these days, the students started or ended wearing the pedometers, and thus recordings are not available for the full day. Students also kept a diary of their daily activities, including the duration of each activity and whether or not the pedometer was worn (available at: http://www.REALKidsAlberta.ca). Trained evaluation assistants returned to schools on the ninth day to collect pedometers and download data to computers. Pedometer recordings were stratified into school day (Monday-Friday) and weekend day (Saturday, Sunday, and holidays) categories.

### Confounding variables

Evaluation assistants measured children’s standing height to the nearest 0.1 cm using stadiometers (Seca-Stadiometer, Germany) and body weight to the nearest 0.1 kg on calibrated digital scales (Health-o-meter, IL, USA). Children removed their shoes for both measurements. Body Mass Index (BMI) was calculated as kg/m^2^. Overweight was defined using the International Obesity Task Force BMI cut-off point established for children and youth [20]. The cut-off point is based on the health-related adult definition of overweight (BMI ≥ 25), but is adjusted to specific age and sex categories for children. Analyses were adjusted for the confounding potential of parental educational attainment, household income, and year of data collection.

### Data processing

For our analyses we only considered pedometer recordings when worn for a minimum of 8 hours per day [21]. Additionally, we required pedometer recordings on at least two school days and one weekend day (weekend and/or holiday) [22]. When these conditions were met, students’ pedometer-measured steps were complemented with step-equivalents of non-ambulatory and non-wear time activities recorded in students’ activity diaries. Briefly, we assigned each activity recoded in activity diaries a youth-specific metabolic equivalent task (MET) unit [23]. Next, we categorized activities by intensity (i.e., moderate, moderate-to-vigorous, vigorous) [24,25] and assigned a step per minute value to each category [26]. Adult METs units were used when youth specific values were not available [24]. When students forgot to wear their pedometer and complete their activity diary, we imputed information from the same hour (s) on other randomly selected valid days. Steps were only imputed within an individual and within school days and weekend days. This method of imputation has been shown to replace data more accurately than traditional group-centered methods that replace missing data with the group mean [27]. These procedures are described in further detail elsewhere [3].

In 2009 and 2011 combined, we had information of pedometer step counts and parental reporting of physical activity-related beliefs and support for 717 girls and 638 boys.

### Data analyses

T-tests and chi-square tests were used to test for data collection year and sex differences. Because observations of students are clustered within schools, we applied random effects models with students nested in schools. For each of the parent belief and support variables, we first applied univariable linear regression models to determine their associations with girls’ and boys’ step-counts taken on weekend days and on school days. Second, we applied multivariable linear regression models to adjust for the confounding potential of parental educational attainment, household income, and year of data collection (referred to as Model 1). Last, we considered parent beliefs and support variables simultaneously while adjusting for the above confounders to quantify their independent importance for children’s step-counts (referred to as Model 2). For each of the parent belief and support variables we used the middle response category as the reference group for analyses.

In a combined analysis, we used an interaction term (defined as the product of school day/weekend day and the parent belief and support variables) in the adjusted linear regression models to quantify the differential effect of beliefs and support on physical activity during school days versus weekend days.

Missing values for parental education attainment and household income were treated as separate covariate categories however we do not present their estimated values. We used Stata Version 12 (Stata Corp, TX USA) to perform the statistical analyses.

## Results

Participant characteristics are reported in Table 1. The average daily step count was higher on school days than on weekend days (12,868 ± 4006 vs. 11763 ± 6636 steps/day p < 0.001). Boys achieved significantly more steps per day than girls on school days (13844 ± 4424 vs. 12000 ± 3366 p < 0.001) and on weekend days (12716 ± 7488 vs. 10914 ± 5645 p < 0.001). Parents also reported to encourage boys to be physically active significantly more than girls (p < 0.001). There were no statistically significant differences in the data collected in 2009 and 2011.

**Table 1:** Characteristics of grade five students participating in the study

	**Mean ± SD/Prevalence**	
	**Girls (n = 717)**	**Boys (n = 638)**
Age	10.9 ± 0.4	10.9 ± 0.4
BMI of child^*^	19.1 ± 3.5	19.7 ± 4.4
Overweight (%)^*^	29.2	34.6
Obese (%)^*^	7.5	10.8
Physical activity^1^		
School day^*^	12,000 ± 3,366	13,844 ± 4,424
Non-school day^*^	10,914 ± 5,645	12,716 ± 7,488
Parent cares about staying fit and exercising		
A little bit	21.7	19.1
Quite a lot	43.4	45.9
Very much	34.9	35.0
Parent encourages their child to be physical activity		
A little bit	13.6	11.3
Quite a lot	44.1	38.9
Very much	42.4	49.8
Parent engages in physical activity with their child^*^		
<1 time/week	40.3	38.8
1–3 times/week	49.0	50.5
≥4 times/week	10.7	10.7
Parental education attainment		
Secondary or less	26.7	22.4
College	42.0	40.5
University and graduate	31.3	37.1
Household income ($)		
<50,000	21.5	19.2
50,001–75,000	15.9	14.3
75,001–100,000	17.4	17.3
>100,001	45.3	49.3

^1^– pedometer-measured steps adjusted for non-ambulatory activities, non-wear time activities, and missing data.

^*^– considered statistically significant at P < 0.05.

### Girls

On school days, increased parental encouragement of physical activity, and increased parental engagement in physical activity together were significantly and positively associated with girls’ daily step counts (Table 2: Model 1). Girls whose parents encouraged physical activity “very much” took an additional 632 (95% CI: 108, 1155) steps per day on school days relative to girls whose parents encouraged them “quite a lot”. Additionally, on school days, girls whose parents engaged in physical activity with them more than four times per week achieved an additional 890 (95% CI: 67, 1712) steps per day relative to girls whose parents engaged in physical activity with them one to three times per week. On weekend days, increased parental encouragement of physical activity was the only positive association with girls’ daily steps counts (β = 997, 95% CI: 130, 1864) that appeared to be statistically significant. Girls whose parents encouraged them “very much” to be physically active took an additional 997 (95% CI: 130, 1864) steps per day on weekend days than girls whose parents encouraged them “quite a lot”. Model 2 of Table 2 reveals that the three parental behaviours (parental care for staying fit and exercising, parental encouragement and parental engagement) are correlated such that none of the three behaviours has a statistically significant effect on girls’ step counts over and above that of the other two behaviours.

**Table 2:** **The association (beta coefficient and 95% confidence interval) of parent belief and support with grade five girls’ physical activity**^**1 **^**on school days and weekend days**

	**School days**						**Weekend days**					
	**Univariable**		**Multivariable**				**Univariable**		**Multivariable**			
			**Model 1**^**2**^		**Model 2**^**3**^				**Model 1**^**2**^		**Model 2**^**3**^	
	**β**	**(95% CI)**	**β**	**(95% CI)**	**β**	**(95% CI)**	**β**	**(95% CI)**	**β**	**(95% CI)**	**β**	**(95% CI)**
Care staying fit & exercising												
A little bit	-506	-1151, 140	-416	-1057, 224	-238	-896, 421	-745	-1806, 316	-605	-1665, 454	-471	-1561, 619
Quite a lot^4^	0	-	0	-	0	-	0	-	0	-	0	-
Very much	347	-214, 907	339	-218, 897	34	-576, 644	605	-315, 1526	613	-309, 1535	170	-839, 1179
Encourage physical activity												
A little bit	-643	-1408, 122	-620	-1380, 140	-503	-1291, 285	-719	-1977, 539	-675	-1932, 582	-639	-1942, 665
Quite a lot^4^	0	-	0	-	0	-	0	-	0	-	0	-
Very much	616^*^	89, 1143	632^*^	108, 1155	546	-33, 1126	970^*^	101, 1839	997^*^	130, 1864	856	-103, 1816
Engage in physical activity												
<1 time/week	-8	-531, 515	19	-497, 535	222	-307, 752	409	-448, 1267	450	-402, 1303	652	-224, 1529
1-3 times/week^4^	0	-	0	-	0	-	0	-	0	-	0	-
>4 time/week	727^*^	-103, 1558	890^*^	67, 1712	712	-120, 1543	942	-420, 2304	1161	-198, 2519	806	-571, 2183

^1^– pedometer-measured steps adjusted for non-ambulatory activities, non-wear time activities, and missing data.

^2^– Model 1 is adjusted for household income, parental educational attainment and year of data collection.

^3^– Model 2 is adjusted for parental care about staying fit and exercising, encourage physical activity, engage in physical activity together, household income, parental educational attainment, and year of data collection.

^4^– reference category.

^*^– considered statistically significant at P < 0.05.

### Boys

On school days, increased encouragement of physical activity was associated with daily step counts. This association was statistically significant (Table 3). Boys whose parents encouraged physical activity “very much” took an additional 1373 (95% CI: 606, 2139) steps per day on school days relative to boys whose parents encouraged physical activity “quite a lot”. This association was independent of parental care for staying fit and exercising and parental engagement in physical activity (Table 3: Multivariable Model 2). On weekend days, increased parental care about staying fit and exercising was positively associated with boys’ daily step counts (Table 3). Boys whose parents reported to care “very much” about staying fit and exercising took an additional 1381 (95% CI: 85, 2676) (Table 3) steps per day on weekend days relative to boys whose parents reported to care “quite a lot”. Also, on weekend days, decreased parental engagement in physical activity was negatively associated with boys’ daily step counts (Table 3). That is, boys whose parents engaged in physical activity with them less than once per week took 1367 fewer steps per day on weekend days relative to boys whose parents engaged in physical activity with them one to three times per week (95% CI: -2643, -90). This association was independent of parental care for staying fit and exercising and parental encouragement (Table 3: Multivariable Model 2).

**Table 3:** **The association (beta coefficient and 95% confidence interval) of parent belief and support with grade five boys’ physical activity**^**1 **^**on school days and weekend days**

	**School days**						**Weekend days**					
	**Univariable**		**Multivariable**				**Univariable**		**Multivariable**			
			**Model 1**^**2**^		**Model 2**^**3**^				**Model 1**^**2**^		**Model 2**^**3**^	
	**β**	**(95% CI)**	**β**	**(95% CI)**	**β**	**(95% CI)**	**β**	**(95% CI)**	**β**	**(95% CI)**	**β**	**(95% CI)**
Care staying fit & exercising												
A little bit	-618	-1542, 306	-654	-1582, 273	-430	-1394, 534	-505	-2105, 1095	-658	-2225, 910	-168	-1811, 1475
Quite a lot^4^	0	-	0	-	0	-	0	-	0	-	0	-
Very much	505	-260, 1270	453	-316, 1222	79	-718, 877	1439^*^	121, 2758	1381^*^	85, 2676	1306	-50, 2661
Encourage physical activity												
A little bit	-269	-1418, 880	-286	-1436, 864	-165	-1358, 1028	-944	-2940, 1051	-1032	-2980, 917	-563	-2570, 1445
Quite a lot^4^	0	-	0	-	0	-	0	-	0	-	0	-
Very much	1408^*^	690, 2126	1372^*^	653, 2092	1373^*^	606, 2139	939	-322, 2201	936	-300, 2171	625	-686, 1935
Engage in physical activity												
<1 time/week	-240	-971, 491	-246	-973, 482	112	-641, 865	-1680^*^	-2937, -424	-1669^*^	-2888, -450	-1367^*^	-2643, -90
1-3 times/week^4^	0	-	0	-	0	-	0	-	0	-	0	-
>4 time/week	492	-650, 1635	566	-571, 1702	264	-882, 1410	-1276	-3254, 701	-1076	-2998, 846	-1350	-3309, 608

^1^– pedometer-measured steps adjusted for non-ambulatory activities, non-wear time activities, and missing data.

^2^– Model 1 is adjusted for household income, parental educational attainment and year of data collection.

^3^– Model 2 is adjusted for parental care about staying fit and exercising, encourage physical activity, engage in physical activity together, household income, parental educational attainment, and year of data collection.

^4^– reference category.

^*^– considered statistically significant at P < 0.05.

There were no significant interactions between parental care for staying fit and exercising, parental encouragement of physical activity, or engagement in physical activity together and girls’ “school day/weekend day” step counts. Parental engagement in physical activity and boys’ “school day/weekend day” physical activity was the single statistically significant interaction. This interaction remained significant after adjusting for potential confounders. Boys whose parents engaged in physical activity with them less than once per week took 1475 (95% CI: -2609, -341) fewer steps per day on weekend days than they did on school days.

## Discussion

This study demonstrates the importance of parental beliefs and support for boys’ and girls’ physical activity on school days and on weekend days. This study demonstrates that parental beliefs and support are important targets for prevention strategies to increase children’s physical activity, which is particularly relevant for weekend days, as children’s activity levels appear to be low during this window of time.

We confirmed children’s physical activity levels to be lower on weekend days than on school days [28-32]. In addition, we observed that parental beliefs and support are positively associated with boys’ and girls’ physical activity achieved on weekend days. For example, we found that girls whose parents reported to encourage physical activity “very much” were significantly more active on weekend days than girls whose parents reported to encourage physical activity “quite a lot”. Similarly, we found that boys whose parents reported to care “very much” about staying fit and exercising were significantly more active on weekend days than boys whose parents reported to care “quite a lot”. To our knowledge, this had not been shown in the literature. These results suggest that specifically targeting parents to encourage and support their child’s physical activity behaviour may be an effective strategy to improve physical activity.

The associations between parental beliefs and support and weekend day physical activity were distinct for boys and girls. For example, parental encouragement was positively associated with girls’ weekend physical activity (Model 1) while parental care for staying fit and exercising was positively associated with boy’s weekend physical activity (Model 1). We observed that associations tended to be stronger among boys than girls. McGuire et al. [33] also found that parental-adolescent relationships were stronger among boys than girls. Further, we observed that parents reported to encourage boys to be physically active significantly more than they encouraged girls. Trost et al. [34] found that parents reported significantly higher levels of support and perceived importance for boys’ physical activity compared to girls’ physical activity. This suggests the importance of health promotion messages that are specific for girls and boys [14], and that educate parents on the importance of physical activity for both boys and girls. Community-based physical activity programs occurring on weekends that involve children and their parents may help to increase boys’ and girls’ physical activity levels on weekends. Health promotion messages should also consider targeting parenting practices as they relate to encouragement to educate parents on how to effectively support their daughters’ activity-related behaviours as girls’ activity levels lag behind that of boys.

Among boys on the weekend, we found that boys whose parents reported to engage in physical activities with them more than four times per week were less active than boys whose parents reported to engage in activities with them between one and three times per week. This is not supported in the literature; others report positive associations between parental engagement in activities and children’s physical activity [6,8-11]. The finding we report here is counter intuitive and may be a result of reverse causation, meaning these parents have recognized their sons to be in a less active subgroup and are intervening in an attempt to raise their activity levels. This seems consistent with our earlier observations, though in a different sample of children, where parents engaged more in activity with their overweight daughters or sons than with their normal weight children [11]. This is an interesting point and warrants further investigation.

Strengths of our study include the use of time-stamped pedometers, a large sample size, and high participation rates for school-based research [35]. A further strength of our study is the adjustments made to raw pedometer-measured steps from activities recorded by students in daily activity logs. There are a few limitations however, that should be acknowledged. Although selected from a population-based sample, the sample of students in this study is not representative of the Alberta population. As such, caution is warranted when generalizing the present results. The cross-sectional design is a limitation and necessitates caution with respect to interpretations of directionality and causality. Furthermore, while the pedometer used in this study has been validated among adults under various conditions [17-19], it has not specifically been validated among 10–11 year-old children. However, because all children wore the same pedometer it is unlikely that this influenced the observed effect size [36]. Also, self-report measures are prone to bias and may produce socially desirable responses to questions surrounding parental beliefs and support. This limitation is acknowledged in the literature [37]. Additionally, given that we did not quantify “how much” parents encourage their child to be active, it is possible that broader parenting practices or styles were captured rather than the actual encouragement itself [38]. This warrants further investigation. To better inform health promotion messages and interventions, future studies may also consider assessing differences in the provisions of encouragement, engagement, and care between boys and girls.

## Conclusions

We showed that parental beliefs and support for physical activity are associated with children’s physical activity on school days and on weekend days. Health promotion strategies and programs that educate parents on how to effectively support their child in developing an active lifestyle may contribute to increasing physical activity levels.

## Abbreviations

REAL Kids Alberta: Raising healthy eating active living kids Alberta.

## Competing interests

The authors declare that they have no competing interests.

## Authors’ contributions

KV assisted with data collection, analyzed and interpreted the data, and drafted the manuscript. SK, KM, JM, BW interpreted the data and critically revised the manuscript. PV conceived and designed the study, interpreted the data, and critically revised the manuscript. All authors read and approved the final version.

## Pre-publication history

The pre-publication history for this paper can be accessed here:

http://www.biomedcentral.com/1471-2458/13/1132/prepub
